# Prediction of 11-year incidence of psychophysically dependent status or death among community-dwelling younger elderlies: from an age-specified community-based cohort study (the NISSIN project)

**DOI:** 10.1186/s12199-021-00968-8

**Published:** 2021-04-10

**Authors:** Satoe Okabayashi, Takashi Kawamura, Hisashi Noma, Kenji Wakai, Masahiko Ando, Kazuyo Tsushita, Hideki Ohira, Shigekazu Ukawa, Akiko Tamakoshi

**Affiliations:** 1 Kyoto University Health Service, Yoshida-Honmachi, Sakyo-ku, Kyoto, 606-8501 Japan; 2 Department of Data Science, The Institute of Statistical Mathematics, 10-3 Midori-cho, Tachikawa, Tokyo, 190-8562 Japan; 3 Department of Preventive Medicine, Nagoya University Graduate School of Medicine, 65 Tsurumai-cho, Showa-ku, Nagoya, 466-8550 Japan; 4 Center for Advanced Medicine and Clinical Research, Nagoya University Hospital, 65, Tsurumai-cho, Showa-ku, Nagoya, 466-8550 Japan; 5 Kagawa Nutrition University, 3-9-21 Chiyoda, Sakado city, Saitama 350-0288 Japan; 6 Department of Psychology, Graduate School of Informatics, Nagoya University, Furo-cho, Chikusa-ku, Nagoya, 464-8601 Japan; 7 Research Unit of Advanced Interdisciplinary Care Science, Graduate School of Human Life Science, Osaka City University, 3-3-138, Sugimoto, Osaka, Sumiyoshi-ku 558-8585 Japan; 8 Department of Public Health, Faculty of Medicine and Graduate School of Medicine, Hokkaido University, North 15, West 7, Kita-ku, Sapporo, 060-8638 Japan

**Keywords:** Aged, Cohort studies, Death, Forecasting, Nursing care

## Abstract

**Background:**

Predicting adverse health events and implementing preventative measures are a necessary challenge. It is important for healthcare planners and policymakers to allocate the limited resource to high-risk persons. Prediction is also important for older individuals, their family members, and clinicians to prepare mentally and financially. The aim of this study is to develop a prediction model for within 11-year dependent status requiring long-term nursing care or death in older adults for each sex.

**Methods:**

We carried out age-specified cohort study of community dwellers in Nisshin City, Japan. The older adults aged 64 years who underwent medical check-up between 1996 and 2000 were included in the study. The primary outcome was the incidence of the psychophysically dependent status or death or by the end of the year of age 75 years. Univariable logistic regression analyses were performed to assess the associations between candidate predictors and the outcome. Using the variables with *p*-values less than 0.1, multivariable logistic regression analyses were then performed with backward stepwise elimination to determine the final predictors for the model.

**Results:**

Of the 1525 female participants at baseline, 105 had an incidence of the study outcome. The final prediction model consisted of 15 variables, and the *c*-statistics for predicting the outcome was 0.763 (95% confidence interval [CI] 0.714–0.813). Of the 1548 male participants at baseline, 211 had incidence of the study outcome. The final prediction model consisted of 16 variables, and the *c*-statistics for predicting the outcome was 0.735 (95% CI 0.699–0.771).

**Conclusions:**

We developed a prediction model for older adults to forecast 11-year incidence of dependent status requiring nursing care or death in each sex. The predictability was fair, but we could not evaluate the external validity of this model. It could be of some help for healthcare planners, policy makers, clinicians, older individuals, and their family members to weigh the priority of support.

**Supplementary Information:**

The online version contains supplementary material available at 10.1186/s12199-021-00968-8.

## Background

Aging is one of the most urgent and important issues in the world. Life expectancy at birth is estimated to increase from 71.9 years in 2015–2020 to 76.9 years in 2045–2050 [1], and the estimated proportion of people over 65 years of age will increase from 8.3% in 2015 to 17.8% in 2060 globally [1]. Aging in Japan is no exception. The proportion of people over 65 years of age is rapidly increasing year by year and will reach 30% of the total population by 2025 [2]. Because such a rapid increase in life expectancy has not been experienced until now, accommodation to the situation is important to provide better support to older individuals.

Predicting adverse health events and implementing preventative measures are a necessary challenge [3]. It is important for healthcare planners and policymakers to allocate the limited resource to high-risk persons [4]. Prediction is also important for older individuals, their family members, and clinicians to prepare mentally and financially [4, 5].

Prediction models for dysfunction or mortality among community-dwelling older individuals using health indicators have been previously reported [6–9]. A prediction scale to anticipate one’s dependent status requiring long-term nursing care was also developed in Japan [10, 11]. However, previous models had two structural limitations. First, these models were constructed based on adults across a wide age range at baseline, where age was the overwhelming factor for mortality prediction compared with any other factors in older adults. In addition, the risk factors were sometimes different between older adults and younger adults [7]. Second, regarding the models predicting one’s dependent status, only a single common model was constructed for both men and women. Since there are many gender differences in risk factors for mortality and adverse health events [12, 13], then, a prediction model at the specified age in each sex is needed.

Most Japanese employees retire from their jobs before 65 years of age, and this makes their big lifestyle change. In addition, the death rate accelerates after 65 years of age [14], even in Japan, a country with the highest rate of longevity [15, 16]. Therefore, the purpose of this study is to develop a model for each sex to predict within 11-year dependent status requiring long-term nursing care or death in older adults using comprehensive medical data from check-ups at age 64 years, one of the key ages for one’s life.

## Methods

### Study population

The study population was extracted from the New Integrated Suburban Seniority Investigation (NISSIN) project, a community-based prospective cohort study of Japanese adults of the specified age. Project rationale and design are described elsewhere [17]. Residents of Nisshin City in Aichi Prefecture, Japan, who were 64 years of age on January 1 of the respective years from 1996 through 2005, were invited to undergo a free-of-charge comprehensive medical check-up [17].

A total of 3073 participants (1548 men and 1525 women, 43.9% of the eligible community residents) who provided informed consent to the study were enrolled in the cohort. They were further invited to undergo the comprehensive medical check-up at age 70 years from 2002 through 2011. The participants have been followed by the second medical check-up, home visits by the municipal public health nurses, the public insurance system for long-term nursing care, or the vital statistics. The subjects of this study were those who participated in the medical check-ups and did not require any long-term nursing care age at 64 years.

### Health check-ups

The health check-up data consisted of laboratory testing and a self-administrated questionnaire. The laboratory testing included physique (height and weight), blood pressure, and blood tests (blood count, liver function, kidney function, and glucose and lipid metabolism). The self-administered questionnaire included their present illnesses, past medical history, family medical history, competence in daily living (Tokyo Metropolitan Institute of Gerontology Index of Competence [TMIG-IC]) [18], depressive tendency (shorter version of the Geriatric Depression Scale [GDS]) [19, 20], mental stress or strain, life satisfaction status (life satisfaction index-K) [17, 21], and lifestyles (eating habits, alcoholic beverage intake, smoking habits, physical activity, sleeping habits, current work, level of participation in community activities, highest level of education, marital status, number of family members living together, and living place). The number of pregnancies, deliveries, and abortions; age at menarche and menopause; birth control use; and hormone replacement therapy-related questions were asked only to women.

### Long-term nursing care in Japan

The government of Japan implemented a public insurance system for long-term nursing care in 2000 for general citizens aged 65 years or more and handicapped citizens aged 40 years or more [22]. When an insured person applies to use any nursing service, they are graded into support-demand level 1 to 2 or care-demand level 1 to 5 by the municipal board assessing the physical and mental conditions [22]. Here, support level 1 is the lowest, and care level 5 is the highest for nursing care. To those in care level 2 or higher who were deemed to need nursing care, ≥ 50-minute support of daily activities such as bathing, defecation, cloth changing, and feeding, or medical treatment (drug administration, tracheal suctioning, and treatment for pressure sores) is provided at the designated frequency.

### Predictors and outcomes

The predictor variables were selected from the data from the health check-ups at age 64 years. The outcome variable was the incidence of the psychophysically dependent status designated as care demand level 2 or higher of the public insurance system (composite outcome) or death of any cause by the end of the year of age 75 years. Outcomes were obtained from the vital statistics or through the insurance system.

### Statistical analyses

We constructed a prediction model in each sex separately. The participants who moved out from Nisshin City without changing to a dependent status of care demand level 2 or more by the end of the year of age 75 years were excluded from the analyses.

In order to develop the prediction model, we first examined the association between each predictor and the outcome using a univariable logistic regression model. Cutoff points of the respective continuous variables were determined by the Youden Index [23]. Nominal and ordinal variables were substituted in the model as the dummy variable of their original categories. Variables with a *p*-value ≤ 0.1 were prescribed, but the variables considered clinically irrelevant were excluded from the model. Variables with three or more categories were binarized after checking their dose-response relationship. The multivariable prediction models were then constructed by backward stepwise elimination to determine the final predictors for the more parsimonious model. The receiver-operating characteristic (ROC) curves were drawn, and the *c*-statistics were calculated. In addition, calibrations for the prediction models were conducted using the calibration plot and the Hosmer-Lemeshow test. Finally, we performed Harrell’s bootstrap bias corrections to adjust for the optimisms of the discriminant measures of the prediction models via 3600 bootstrap resampling [24].

Statistical analyses were conducted using STATA 15.0 (StataCop, College Station, TX, USA) and R ver. 3.5.1 (R Foundation for Statistical Computing, Vienna, Austria).

### Ethical considerations

For informed consent, an opt-out approach was used from 1996 through 2001, and individual written informed consent was obtained thereafter [17, 25]. The study was approved by the Ethics Committees of Nagoya University Graduate School of Medicine, the National Center for Geriatrics and Gerontology of Japan, Aichi Medical University, and Hokkaido University Graduate School of Medicine.

## Results

### Women

Of the 1525 female participants in the medical check-up at age 64 years, we excluded 106 women who moved out without becoming legal dependents by the end of age 75 years from the analyses. Baseline characteristics of the participants are shown in Table 1. Less than 5% of the female participants had a medical history of severe diseases such as cancer and myocardial infarction. Their competence of daily living was relatively good based on the TMIG score [26]. Among the 1419 eligible female participants, 105 had an incidence of the outcome variables (60 died and 45 developed a dependent living status).

**Table 1:** Baseline characteristics of the eligible study participants

	Women		Men	
	All participants	Missing	All participants	Missing
	*n*=1419		*n*=1445	
Present illness				
Hypertension, *n* (%)	551 (38.8)	0 (0.0)	729 (50.4)	0 (0.0)
Hyperlipidemia, *n* (%)	915 (64.5)	0 (0.0)	791 (54.7)	1 (0.07)
Diabetes mellitus, *n* (%)	106 (7.5)	0 (0.0)	205 (14.2)	2 (0.14)
Past medical history				
Cancer, *n* (%)	61 (4.3)	0 (0.0)	47 (3.3)	0 (0.0)
Myocardial infarction, *n* (%)	9 (0.63)	0 (0.0)	32 (2.2)	0 (0.0)
Stroke, *n* (%)	34 (2.4)	0 (0.0)	56 (3.9)	0 (0.0)
Hepatitis, *n* (%)	41 (2.9)	0 (0.0)	77 (5.3)	0 (0.0)
TMIG, median (IQR)	13 (12, 13)	8 (0.56)	12 (11, 13)	6 (0.42)
Depressive mood,^a^ median (IQR)	4 (2, 5)	10 (0.70)	3 (1, 5)	9 (0.62)
Life satisfaction,^b^ median (IQR)	5 (4, 7)	9 (0.63)	5 (4, 7)	10 (0.69)
Smoking, *n* (%)		0 (0.0)		1 (0.07)
Current	48 (3.4)		457 (31.6)	
Past	68 (4.8)		704 (48.7)	
Never	1303 (91.8)		283 (19.6)	
Alcoholic beverage intake (yes), *n* (%)	284 (20.0)	0 (0.0)	990 (68.5)	1 (0.0)
Physical exercise, *n* (%)		1 (0.07)		2 (0.14)
Seldom	599 (42.2)		567 (39.2)	
<1/week	112 (7.9)		146 (10.1)	
≥1/week	707 (49.8)		730 (50.5)	
Final education		12 (0.85)		6 (0.42)
Junior high school	506 (35.7)		415 (28.7)	
Senior high school	679 (47.9)		561 (38.8)	
Junior college or higher	222 (15.6)		463 (32.0)	
Marital status		12 (0.85)		4 (0.28)
Presently married	1181 (83.2)		1374 (95.1)	
Never, widowed, or divorced	226 (15.9)		67 (4.6)	

*TMIG* Tokyo Metropolitan Institute of Gerontology Index of Competence, *IQR* Interquartile range

^a^The score of Geriatric Depression Scale

^b^The score of Life Satisfaction Index-K

Risk or preventive factors associated with the outcome in univariable logistic regression analyses with *p* ≤0.1 included 27 variables (Supplementary table 1). The tentative risk factors included the test result of systolic hypertension, erythrocytosis, higher alanine transaminase (ALT) level, higher serum creatinine level, and hypertriglyceridemia; a present diagnosis of diabetes mellitus; past history of otolaryngological diseases, stroke, hepatitis, and arthritis including rheumatoid arthritis; and lifestyles of alcoholic beverage intake, current smoking, skipping breakfast and midnight snack eating, preference of salty tastes, a slow gait, longer daily sleeping, and frequent night-time awakening. The tentative protective factors included higher body mass index (BMI), past history of cystitis, higher TMIG-IC, life satisfaction, full/various amount of eating, every day/often snacking, routine exercise, and engagement in a hobby group.

**Fig. 1:**
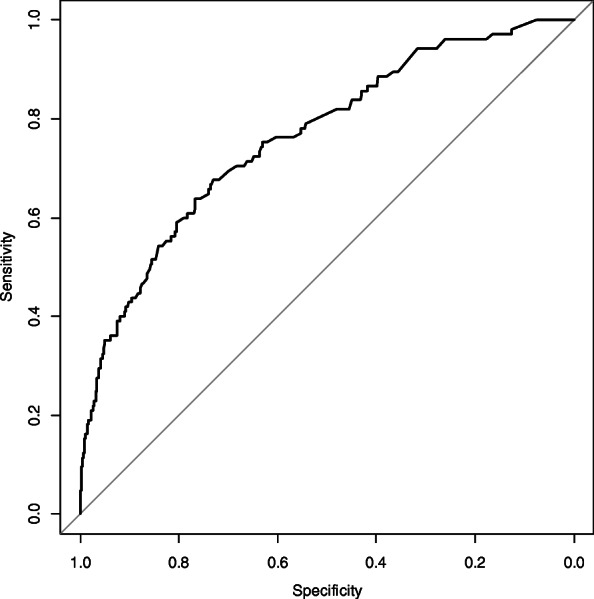
The receiver-operating characteristic curve of the model to estimate the probability of dependent status/death (women)

The final multivariable prediction model consisted of 15 variables with *p* < 0.05, including several items from the test results (BMI ≥ 22.6 kg/m^2^, systolic blood pressure ≥ 156 mm Hg, hemoglobin ≥ 14.5 mg/dl, ALT ≥ 35.0 U/ml), past medical history (otolaryngological diseases, stroke, and cystitis), a family medical history of hypertension, life satisfaction index-K ≥ 4, and some lifestyles (eating a full or moderate amount, alcoholic beverage intake, current smoking, slow gait, night-time awakening ≥ 3 times, sleeping more than 8 h a day) (Table 2). The *c*-statistics for predicting the outcome was 0.763 (95% confidence interval [CI] 0.714–0.813) (Fig. 1), and the final model had a satisfactory goodness of fit (Hosmer-Lemeshow statistic *p* = 0.93). The optimism-adjusted *c*-statistics was 0.734 (95% CI 0.685–0.784).

**Table 2:** Predictors for dependent status or death for the final prediction model (women)

	Subjects	Outcome^a^	(%)	*β*-coefficient	OR	95% CI
Laboratory testing						
Physique						
BMI ≥22.6	688	41	(6.0)	−0.81	0.44	0.28–0.70
Systolic blood pressure ≥156 mm Hg	136	23	(16.9)	1.19	3.30	1.88–5.79
Blood test						
Hemoglobin ≥14.5 mg/dl	62	11	(17.7)	1.17	3.21	1.50–6.89
ALT ≥35.0 U/ml	82	11	(13.4)	0.77	2.16	1.03–4.52
Questionnaire						
Past medical history						
Otolaryngological diseases	180	20	(11.1)	0.80	2.23	1.27–3.92
Stroke	34	9	(26.5)	1.57	4.79	1.92–11.94
Cystitis	179	6	(3.4)	−0.89	0.41	0.17–0.98
Family medical history						
Hypertension	289	13	(4.5)	−0.68	0.50	0.26–0.96
Life satisfaction index-K ≥4	1069	66	(6.2)	−0.48	0.62	0.39–0.97
Eating full or moderate amount	638	38	(6.0)	−0.43	0.65	0.42–1.02
Alcohol drinker	284	29	(10.2)	0.60	1.82	1.12–2.95
Current smoker	48	10	(20.8)	1.25	3.50	1.56–7.84
Slow gait	196	28	(14.3)	0.97	2.63	1.56–4.45
Night-time awakening ≥3 times	89	15	(16.9)	1.01	2.75	1.45–5.22
Daily sleeping ≥8 h	265	29	(10.9)	0.48	1.62	0.99–2.65
Intercept				−3.16		

*BMI* Body mass index, *ALT* Alanine transaminase, *OR* Odds ratio, *CI* Confidence interval

^a^Death or dependent status of care demand level 2 or more

### Men

Of the 1548 male participants in the medical check-up at age 64 years, we excluded 103 men who moved out without becoming legal dependents by the end of age 75 years from the analyses. Baseline characteristics of the participants are shown in Table 1. Less than 5% of the male participants had a medical history of severe diseases such as cancer and myocardial infarction. Their competence of daily living was relatively good based on TMIG score [26]. Among the 1445 eligible subjects, 211 had an incidence of the outcome variables (172 died and 39 developed a dependent status).

Risk or preventive factors associated with the outcome in univariable logistic regression analyses with *p* ≤ 0.1 included 36 variables (Supplementary table 2). The tentative risk factors included the test results of systolic and diastolic hypertension, higher ALT level, and higher serum creatinine level; a present diagnosis of diabetes mellitus; a past history of pulmonary tuberculosis or pleurisy, chronic bronchitis, hemorrhagic stroke, and hepatitis; a family medical history of stroke; depressive tendency; and lifestyles of frequent eating of snack/midnight snack, frequent eating dinner alone, preference of oily dishes, dietary restrictions by a physician, a slow gait, shorter walking time, daily car using, long time to fall asleep, current smoking, passive smoking, low level of education, unmarried status, living in residential/agricultural area, and shorter duration of living in the place. The tentative protective factors included erythrocytosis and hyper high-density lipoprotein cholesterolemia; a past history of gastroduodenal polyp; a family medical history of cancer or hypertension; life satisfaction; irregular dinner time; frequent eating out for lunch; preference of salty tastes; routine exercise; and participation in community activities (community events, visiting friends or relatives living in neighborhood).

**Fig. 2:**
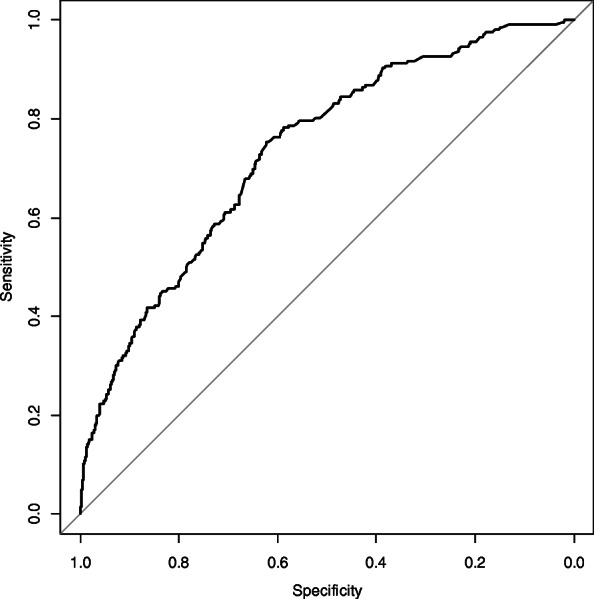
The receiver-operating characteristic curve of the model to estimate the probability of dependent status/death (men)

The final model consisted of 16 variables with *p* < 0.05, including three items from the test results (systolic blood pressure ≥ 130 mm Hg, ALT ≥ 29.0 U/ml, serum creatinine ≥ 0.9 mg/dl), a present diagnosis of diabetes mellitus, past medical history (hemorrhagic stroke, and hepatitis), family medical history (hypertension or cancer), and some lifestyles (irregular dinner time, frequently eating snack/midnight snack once per week or more, preference of salty tastes, current smoking, a slow gait, long time to fall asleep [30 min or more], highest education of junior high school or lower, and unmarried status) (Table 3). The *c*-statistics for predicting the outcome variables was 0.735 (95% CI 0.699–0.771) (Fig. 2), and the final model had a satisfactory goodness of fit (Hosmer-Lemeshow statistic *p* = 0.58). The optimism-adjusted *c*-statistics was 0.713 (95% CI 0.677–0.748).

**Table 3:** Predictors for dependent status or death for the final prediction model (men)

	Subjects	Outcome^a^	(%)	*β*-coefficient	OR	95% CI
Laboratory testing						
Physique						
Systolic blood pressure ≥130 mm Hg	972	165	(17.0)	0.61	1.84	1.26–2.67
Blood test						
ALT ≥29.0 U/ml	269	57	(21.2)	0.43	1.54	1.06–2.23
Serum creatinine ≥0.9 mg/dl	729	123	(16.9)	0.46	1.58	1.15–2.18
Questionnaire						
Present illness						
Diabetes mellitus	205	47	(22.9)	0.55	1.74	1.16–2.60
Past medical history						
Hemorrhage stroke	17	7	(41.2)	1.32	3.74	1.27–11.00
Hepatitis	77	21	(27.3)	0.99	2.69	1.51–4.81
Family medical history						
Cancer	560	62	(11.1)	−0.49	0.61	0.44–0.86
Hypertension	261	28	(10.7)	−0.52	0.60	0.38–0.94
Irregular dinner time	1196	186	(15.6)	−0.65	0.52	0.32–0.84
Frequently eating snack/midnight snack once per week or more	584	99	(17.0)	0.38	1.47	1.07–2.02
Preference of salty tastes	1302	180	(13.8)	−0.69	0.50	0.31–0.80
Current smoker	457	94	(20.6)	0.72	2.05	1.48–2.84
Slow gait	175	44	(25.1)	0.69	1.99	1.31–3.03
Time to fall asleep (≥30 min)	175	38	(21.7)	0.48	1.62	1.06–2.49
Junior high school or lower level of education	415	78	(18.8)	0.30	1.35	0.97–1.89
Divorced, widowed, or never married	67	19	(28.4)	1.14	3.12	1.73–5.65
Intercept				−2.56		

*ALT* Alanine transaminase, *OR* Odds ratio, *CI* Confidence interval

^a^Death and dependent status of care demand level 2 or more

## Discussion

We developed and validated prediction models for 11-year incidence of dependent status or death in each sex using comprehensive health check-up data and detailed self-administered questionnaire answers. To our knowledge, this is the first prediction model to forecast the prognosis of older adults, separated by sex. These models were fairly calibrated and predictive with a *c*-statistic of 0.763 in women and 0.748 in men. This model will be referred for the allocation of the resources to high-risk persons if the model will be spreading use in the society. The older adults using this model can also prepare for their future based on the result.

The strong points of this study are age and sex. First, all the participants were the same age, 64 years old at baseline. By focusing the subjects’ age at 64 years, we could overcome the limitation of the previous studies that the age was widely distributed which strongly affected the incidence of outcomes. In addition to this, age of 64 years is just before a new stage in life such as retirement from the workforce and entrance into pension life. Vital statistics show that mortality and morbidity markedly increase around this age [14]. Adults of this age have to consider and plan for their subsequent life. Second, we constructed the prediction model separately by sex. The biological factors such as hormonal secretion and activity of cytokines [27, 28] and social factors including marital status and healthcare utilization are much different by sex [28, 29]. Therefore, the prediction models constructed separately by sex are valuable.

This is the first long-term (more than 10 years) prediction model focusing on both psychophysically dependent status and death at the same time in community-dwelling older adults. Psychophysically dependent status requiring long-term nursing care is a heavy burden for older adults, their family members, and public health. Furthermore, a prediction focusing only on dependent status or dysfunction would miss the extreme dysfunction, that is death. If a medical treatment that can reduce the morbidity increased the mortality, the treatment would not be acceptable. Therefore, our composite outcome, dependent status or death, is relevant for the prediction model of high-risk persons.

The predictability of our model was similar to the previous models that predict death or dysfunction [6, 7]. In general, age is the dominant factor in such kind of prediction models with the participants of wide age range because the death and dependent status strongly depended on the age [14]. Therefore, the predictability of our model with the participants of the same key age can be substantially good when we compare our model with former models constructed by the participants of wide age range. However, the variables used in our final models were somewhat different to those of previous models. The variables of lifestyle such as eating and sleeping habits were included in our model, which were modifiable by the persons’ will. On the other hand, the variables often included in previous prediction models, such as a medical history of cancer and cardiovascular diseases, were statistically eliminated from our models. We consider that this is because the participants of our study are all community-dwelling 64-year-old people who are younger and healthier than those of previous studies, and the prevalence of these diseases was too low to detect any associations with the study outcome.

Interestingly, social factors such as marital and educational status were well associated with the outcome in men. It has been previously reported that living alone and eating alone would increase mortality, especially in men [30–32]. Marital status was also reported to be associated with frailty [29]. Older men who are divorced/widowed/never married often live alone or eat alone, and these living states might induce unhealthy lifestyle and evoke adverse outcomes. In contrast, a high educational status would lead to a higher pension income, which could help assure routine disease screening and early treatment in men, the typical breadwinner of a family [33].

There are several limitations to the study. First, the dependent status requiring nursing care could be detected since 2000 when the insurance system for long-term nursing care was introduced in Japan. However, the citizens who developed a dependent status requiring long-term nursing care before 2000 were supposed to apply for the care or to die after 2000. Second, the judgment of care level is subjective. However, the standard for designation is made by the expert board in each municipality and expected to be fair and stable. Third, gene and blood substances which were potentially related to one’s prognosis in older adults were not included in our prediction model. Some of them were already examined in our field [34]. However, it is more practical and feasible to construct models using data easily collected in daily practices. Fourth, only 43.9% of the eligible community residents participated in this study, and there must be some selection bias. Not a few residents who did not participate in the health check-up might have serious physical/mental condition or receive heavy medical care. However, we aimed to make a prediction model of dependent status or death for the old individuals without major social disabilities. Then, we guessed the participants would be more representative of our target population than the whole 64-year population. Fifth, this prediction model can be used only for Japanese participants aged 64 years. However, as we mentioned, the age 64 years is the key age of one’s life, and we believe that this model is meaningful. Besides, we could not evaluate the external validity of this model so that external validation study in another cohort is the tasks in the future. Finally, more than 20 years have passed since the baseline data were collected. In particular, the information and communication technology has been tremendously developed in these decades, and the lifestyle of older adults has also been affected by such time trend. However, “out-of-data” is inevitable for long-term cohort studies.

## Conclusion

In conclusion, based on this age-specific community-based cohort study, we have developed and validated a prediction model for older adults to predict 11-year incidences of death or dependent status requiring nursing care in each sex. Our model relies on 15 variables in women and 16 variables in men, which are available by health check-ups and a self-administered questionnaire. The predictive ability was fair in both sexes, and it could help healthcare planners, policymakers, clinicians, older individuals, and their family members to weigh the priority of support in the highly aged seniorities.

## Supplementary Information

**Additional file 1: Supplementary table 1.** Potential predictors for dependent status or death by the end of the year of age 75 years in univariable logistic regression analyses (women)

**Additional file 2: Supplementary table 2.** Potential predictors for dependent status or death by the end of the year of age 75 years in univariable logistic regression analyses (men)

## Data Availability

The data set analyzed for the current study is not publicly available because we did not obtain informed consent from the participants for the data use by persons except the researchers of the NISSIN project.

## Abbreviations

TMIG-IC: Tokyo Metropolitan Institute of Gerontology Index of Competence
GDS: Geriatric Depression Scale
ROC: Receiver-operating characteristic
ALT: Alanine transaminase
BMI: Body mass index
CI: Confidence interval
