# Correction to: Prediction of 11-year incidence of psychophysically dependent status or death among community-dwelling younger elderlies: from an age-specified community-based cohort study (the NISSIN project)

**DOI:** 10.1186/s12199-021-00977-7

**Published:** 2021-05-01

**Authors:** Satoe Okabayashi, Takashi Kawamura, Hisashi Noma, Kenji Wakai, Masahiko Ando, Kazuyo Tsushita, Hideki Ohira, Shigekazu Ukawa, Akiko Tamakoshi

**Affiliations:** 1 Kyoto University Health Service, Yoshida-Honmachi, Sakyo-ku, Kyoto, 606-8501 Japan; 2 Department of Data Science, The Institute of Statistical Mathematics, 10-3 Midori-cho, Tachikawa, Tokyo, 190-8562 Japan; 3 Department of Preventive Medicine, Nagoya University Graduate School of Medicine, 65 Tsurumai-cho, Showa-ku, Nagoya, 466-8550 Japan; 4 Center for Advanced Medicine and Clinical Research, Nagoya University Hospital, 65, Tsurumai-cho, Showa-ku, Nagoya, 466-8550 Japan; 5 Kagawa Nutrition University, 3-9-21 Chiyoda, Sakado city, Saitama, 350-0288 Japan; 6 Department of Psychology, Graduate School of Informatics, Nagoya University, Furo-cho, Chikusa-ku, Nagoya, 464-8601 Japan; 7 Research Unit of Advanced Interdisciplinary Care Science, Graduate School of Human Life Science, Osaka City University, 3-3-138, Sugimoto, Osaka, Sumiyoshi-ku 558-8585 Japan; 8 Department of Public Health, Faculty of Medicine and Graduate School of Medicine, Hokkaido University, North 15, West 7, Kita-ku, Sapporo, 060-8638 Japan

**Correction to: Environ Health Prev Med 26, 45 (2021)**

**https://doi.org/10.1186/s12199-021-00968-8**

Following the publication of the original article [1] the authors noticed a mistake in the second sentence of the Abstract's Method section.

It was written as below.

The older adults aged 64 years who underwent medical check-up between 1996 and **2000** were included in the study.

However, the correct writing is as follows.

The older adults aged 64 years who underwent medical check-up between 1996 and **2005** were included in the study.

The original article [1] has been updated.
